# Assessing the Efficacy of Cognitive-Behavioral Therapy on Body Image in Adolescent Scoliosis Patients Using Virtual Reality

**DOI:** 10.3390/jcm13216422

**Published:** 2024-10-26

**Authors:** Ewa Misterska, Marek Tomaszewski, Filip Górski, Jakub Gapsa, Anna Słysz, Maciej Głowacki

**Affiliations:** 1 Department of Pedagogy and Psychology, University of Security, 60-778 Poznan, Poland; 2 Chair of Psychiatry, Poznan University of Medical Sciences, 60-572 Poznan, Poland; 3 Department of Spine Disorders and Pediatric Orthopedics, Poznan University of Medical Sciences, 61-545 Poznan, Poland; m.tomaszewski@ump.edu.pl; 4 Institute of Materials Technology, Poznan University of Technology, 61-138 Poznan, Poland; filip.gorski@put.poznan.pl (F.G.); jakub.gapsa@put.poznan.pl (J.G.); 5 Department of Psychology and Cognitive Sciences, Adam Mickiewicz University, 60-547 Poznan, Poland; aslysz@amu.edu.pl; 6 Department of Pediatric Orthopedics and Traumatology, Poznan University of Medical Sciences, 61-545 Poznan, Poland; mglowacki@ump.edu.pl

**Keywords:** adolescent idiopathic scoliosis, virtual reality (VR), virtual mirror, cognitive-behavioral therapy, body image

## Abstract

**Background/Objectives:** Adolescents with idiopathic scoliosis require emotional support to change their experience of their desired body shape and to feel optimistic about the cosmetic results of surgical treatment. Recently, the use of virtual reality in psychological assessment and treatment has given specialists a technology that appears particularly well-suited for addressing body image disorders. The study objectives were two-fold. Firstly, we aimed to evaluate changes within the body image of scoliosis patients pre- and postoperatively. Secondly, we aimed to investigate if differences in body image exist in scoliosis females after implementing cognitive-behavioral therapy. **Methods:** Thirty-six total scoliosis patients participated in the 1st and 2nd study phases. The psychotherapy took place before and after surgery and during the patient’s stay in the hospital. Body image was assessed using a virtual reality-based application, “Avatar Scoliosis 3D”. **Results:** Regarding body image dissatisfaction evaluated via virtual tasks, the difference between the desired by patients and actual (based on the radiographic parameters) body shape is significant preoperatively in both scoliosis samples: with and without therapy (*p* < 0.000001 and *p* < 0.000001, respectively). **Conclusions:** The results of the present study may have important implications for developing standards for body image disorder treatments in scoliosis patients. We revealed that irrespective of received therapeutic support, scoliosis patients accurately estimate their body shape pre- and postoperatively, and they feel dissatisfied with their body preoperatively but not postoperatively.

## 1. Introduction

Adolescent idiopathic scoliosis (AIS) is acknowledged as a significant risk factor for psychological disturbances. Upon diagnosis, approximately 40% of patients report isolation, denial, distress, and depression [1,2]. According to Danielsson et al. [3], 49% of patients who had surgery and 34% of those under brace treatment, compared to 15% of control subjects, reported social activity limitations and self-consciousness about their appearance due to their back condition. Adolescents with significant spinal deformities are prone to teasing and self-consciousness, which can lead to body image disturbances. These disturbances might be connected to depression, low self-esteem, social anxiety, and finally, lead to a diminished quality of life [4]. Thus, these adolescents may struggle to form meaningful connections and feel accepted within their peer circles [5].

### 1.1. Adolescent Idiopathic Scoliosis

AIS is indeed the most common type of scoliosis observed in adolescents. It affects approximately 2–3% of children aged 10–16. Girls are more likely to experience progression, with a ratio of 3.6–1 compared to boys [6]. AIS is characterized by a three-dimensional spine deformity involving lateral deviation of the vertebral column accompanied by rotation of the vertebrae and sagittal (referring to the vertical plane that divides the body into left and right sides) spinal curvature disruption [6,7].

As in many other diseases, attention is paid to the genetic determinants of scoliosis [6,8]. AIS is recognized as a chronic disease with consequences, like many other orthopedic diseases [8,9], on many planes. The scoliosis Cobb angle serves as a crucial measure for assessing the severity of the condition. This method involves identifying the upper and lower end vertebrae on an anteroposterior X-Ray of the entire spine. Vertical lines are drawn from the endplate lines of these vertebrae, and the angle between these two lines is defined as the Cobb angle [10].

AIS with a high Cobb angle can significantly diminish a patient’s physical capacity. Moreover, it may contribute to the development of neurological complications and, in severe cases, can result in cardiorespiratory failure and premature mortality [11]. Specifically, AIS with a high Cobb angle has the potential to significantly impair physical capacity, lead to neurological deficits, and, in extreme cases, cause cardiorespiratory failure and premature death [11]. The treatment options in AIS include observation, brace prescription, posture training, reassurance, and surgery [12]. The indications for AIS operative treatment are a steadily increasing angle of curvature up to a 45–50-degree Cobb angle, neurological disorders, and pain, as well as aesthetic reasons connected to rib hump or lumbar curve in some cases [11]. 

The criteria for surgery in AIS include a progressively worsening curvature that reaches a Cobb angle of 45–50 degrees, the presence of neurological disorders and pain, and, in some instances, aesthetic concerns related to a rib hump or lumbar curve [11]. Moreover, patients with progressive AIS may present body deformities, including, e.g., an uneven shoulder level and an asymmetric waist. These easily visible body deformities can hurt an adolescent’s personality development, levels of self-esteem, self-, and body image. [13,14]. Specifically, Auerbach et al. [5] indicated greater scoliosis-related body image issues in AIS patients compared with healthy peers.

Surgery due to AIS often effectively decreases severe deformities and lowers the risk of scoliosis progression [15,16]. However, technical success by the surgeon only sometimes equates to patient satisfaction with the surgery’s outcomes. Patient satisfaction with the surgical results appears to be disconnected from the medical benefits of surgery, which include maintaining pulmonary function and preventing osteoarthritis [17].

However, the cosmesis of the back and shoulders (recognized as a surgical correction of a disfiguring defect), or the cosmetic improvements made by a surgeon, is extremely important to AIS patients. Consequently, patients’ perception of their postoperative silhouette is crucial to their satisfaction with the surgical outcome [18].

### 1.2. Body Representations in AIS Patients 

Body representation disorders refer to neurological or psychological conditions in which an individual’s perception, awareness, or mental representation of their body is distorted or impaired. These disorders can manifest in various ways, such as a distorted sense of body size, shape, or position, and are often associated with conditions like anorexia nervosa (AN), body dysmorphic disorder, or specific neurological impairments [19].

Body representation disorders are significant clinical issues affecting individuals with AIS. However, current research on this topic is insufficient. While it has clearly shown the prevalence of body image disorders, it has not adequately explored body schema alterations. There are no clear associations between these disorders and clinical measures of scoliosis severity (such as radiographic parameters), nor are there standardized assessment and treatment procedures [20]. Many researchers have highlighted the high prevalence of body image dissatisfaction among AIS patients, who consistently score lower in self-image compared to healthy adolescents [21,22]. Furthermore, patients with more severe deformities exhibit greater disturbances in body image compared to those with milder cases [23,24].

When evaluating the impact of various treatment options on body image, surgical treatment provides the most substantial evidence of improvement in self-image. However, it is essential to note that patients who undergo surgery typically have more severe scoliosis, with mean Cobb angles greater than 50° compared to those brace-treated [25,26]. The aesthetic changes postsurgery are more noticeable, thereby significantly influencing self-reported measures of body image perceptions. Despite this, the severity of the deformity does not always correlate with the decision to undergo surgery, indicating possible discrepancies between psychological factors and clinical measures [20].

Some research on body image disorders (BID) in clinical populations, like patients undergoing treatment for AN, has shown that these individuals often incorrectly estimate their body size in both visual size estimation tasks [27,28] and nonvisual assessments [29]. Some authors have also found that women with BID do not perceive their bodies or those of weight-matched individuals differently from healthy controls. Still, they judge what weight is desirable differently [30]. The present study hypothesizes that a similar phenomenon related to body disfigurement perception may occur in adolescents with scoliosis. It is important to highlight that existing findings on body shape evaluation are inconsistent. While body dissatisfaction has been associated with changes in body shape and weight, improvements in these factors do not necessarily lead to a reduction in dissatisfaction with one’s physical appearance [31].

Thus, AIS patients may continue to overestimate body deformities following surgery, including the severity of the rib hump, scapular and rib prominence, uneven shoulders, and asymmetry of the waistline. This may result in persistent disturbances in body image over the long term. Moreover, it remains to be seen under which conditions scoliosis patients might concentrate on a negative body image, and they cannot reset this perception even after significant improvements in body shape postsurgery. Consequently, AIS patients may require emotional support to change their experience of their desired body shape and to feel optimistic about the cosmetic results of scoliosis surgery.

In conclusion, body image dissatisfaction, negative attitudes toward perceived body shape, and body schema alterations should be recognized as highly comorbid conditions in AIS. Therefore, clinicians should consider the multifactorial nature of scoliosis and implement appropriate assessments and treatments alongside traditional methods, such as bracing, surgery, or physiotherapy.

### 1.3. The Assessment of Body Image

Body distortion can be assessed in many ways, but a gold standard has yet to be established. Some assessment methods focus on evaluating distortions of the whole body, while others target specific body sites [32]. However, most existing instruments for measuring body image distortion rely on whole-body techniques [33,34,35,36,37]. Additionally, available questionnaires can assess perceived body awareness, which can be supplemented with drawings depicting perceived disturbances [38,39,40,41,42]. Researchers also evaluate the integrity of body or movement representations by having subjects estimate the size of their body parts, engage in motor imagery, visually recognize body-object interactions, or determine the sequence of segments involved in a particular movement [37].

Additionally, video images and numerical photographs have been incorporated into measures of body image distortion [33,34]. Clinical observations suggest, however, that disturbances in body perception may be more pronounced during movement than in static conditions [42]. Consequently, movement-based assessments could better reflect real-world interactions between the body and its environment, potentially providing new insights into body distortion and its associations with AIS. 

Referring to recent modern assessment tools, in the last decades, the effective use of virtual reality (VR) in psychological assessment and treatment has given specialists a technology that appears particularly well-suited for addressing body image disturbances [43]. Regarding terminological matters, Extended Reality (XR) is a group of digital technologies that enable user interaction with digitally created 3D objects in real time. VR is one of the three core technologies in the group. XR encompasses the entire simulated reality continuum, offering a broad spectrum of applications from fully immersive virtual environments to real-world environments enhanced with virtual elements—augmented reality (AR) and mixed reality (MR). Using the Milgram continuum [44] and newer elaborations [45], it can be stated that VR is as far from the real world as possible, offering complete immersion into a digital world and interacting only with virtual objects, not with real ones, while the other technologies assume some extent of interaction and perception of the real world while working with digital objects. 

Pioneering studies on the application of VR for assessing and treating body image were conducted by Riva et al. [46]. They created a software tool for evaluating body image disturbances called the Body Image Virtual Reality Scale (BIVRS) [47]. The internal consistency of the BIVRS, as measured by Cronbach’s alpha, has been reported in several studies. For example, a survey by Perpiñá et al. [48] on VR in eating disorders reported a Cronbach’s alpha of 0.89 for the BIVRS. They also reported ICC values indicating high stability over time, ensuring that repeated assessments with BIVRS yield similar results. This is crucial for monitoring changes in body image perceptions throughout interventions. They also underscore its reliability as a measurement tool in clinical settings.

Perpiñá et al. [48,49] developed another software application for assessing and treating body image disturbances in individuals with eating disorders. This software features a 3D human figure with adjustable body parts controlled by slider bars on the computer screen. Participants can modify the size of each body part by moving the sliders left or right. Meanwhile, Mölbert et al. [30] used biometrically modeled self-avatars of female adults, comparing them with standardized avatars to study body image differences between patients with AN and healthy controls.

These applications enhance assessment methods based on silhouettes by incorporating a third dimension into the figures presented to the user. As a result, the figures become more realistic, allowing study participants to more easily identify with them [43]. Additionally, these figures can be modified to reflect various aspects of body image, including perceived or ideal body size. Immersive systems like VR glasses or head-mounted displays enable participants to encounter their virtual bodies within the same physical environment and at true-to-life size or shape [43]. This immersive and interactive nature defines VR, fostering a sense of presence where users feel deeply engaged with the virtual environment [46]. From a psychological standpoint, VR offers an added dimension over other synthetic experiences through the sensation of presence—the feeling of being within the digital environment, replacing real-world perceptions [47,50]. In conclusion, VR exposure is a beneficial technology for researching body image distortion and dissatisfaction. 

Considering these advantages, we recently developed the VR application “Avatar Scoliosis 3D”. It is a valuable method to investigate changes within body representation during AIS treatment [51].

“Avatar Scoliosis 3D” system uses a mixed approach, neither fully VR nor AR. It uses the concept of a virtual mirror based on Augmented Reality, but the displayed avatars are purely digital. The closest notion would be nonimmersive VR, as the image is displayed on a large screen (not in goggles), with motion tracking used to stimulate the sense of proprioception in the users (avatars representing their movements in real time).

### 1.4. Cognitive-Behavioral (CBT) Interventions for Body Image Disorders

Koch et al. [52] suggested that AIS females may require emotional support to feel optimistic about the visual effects of scoliosis surgery. There is growing evidence supporting the efficacy of cognitive-behavioral therapy (CBT), being a form of body psychotherapy, in addressing body image disturbances, as highlighted by Koemeda-Lutz et al. [53] and Rohricht et al. [54]. CBT remains one of the most extensively examined and evidence-based treatments for body image disorders. In brief, CBT focuses on changing irrational and harmful thoughts, emotions, and behaviors using methods like self-monitoring, desensitization, psychoeducation, cognitive restructuring, or exposure and response prevention [55,56,57]. Cash et al. were pioneers in creating and assessing CBT programs addressing body image concerns. In 1987, Butters and Cash carried out an RTC to evaluate a therapist-led, individually structured CBT program for college women in the United States who had body image disorders [58]. Additionally, multiple recent experimental studies have proved that CBT is very effective in treating severely negative body images and positively affects other areas of functioning [56].

Furthermore, CBT has proven effective in treating disturbed body image in individuals with body dysmorphic disorder (BDD). Jarry and Ip [59] conducted a meta-analysis, “The Effectiveness of Cognitive-Behavioral Treatment on Body Image”, primarily using university student samples. They concluded that CBT is efficacious in improving disturbed body image. Similarly, Veale et al. reported that CBT has successfully alleviated symptoms of BDD in women [60]. These findings align with results from studies by Rosen et al. [61], Khemlani-Patel et al. [62], and Allen [63], which also demonstrated the effectiveness of CBT in addressing disturbed body image.

Thus, CBT methods could assess and modify some attitudes and emotions connected to body shape before and after the AIS surgical treatment. CBT could involve AIS patients in identifying and discussing appearance-preoccupying rituals, such as mirror checking, and time-consuming efforts to manage or alter appearance through meticulous grooming routines. These therapeutic objectives are typically achieved by integrating various CBT techniques, including countering, alternative interpretation, deactivating presurgical body shape beliefs, or label shifting [55,56,57,58].

### 1.5. Study Objectives

The study objectives were two-fold. Firstly, we aimed to longitudinally evaluate changes within the body image of AIS patients at two time points: preoperatively and postoperatively (within two weeks following surgery). Body image will be assessed using a novel methodology that applies VR-related tasks. Our main hypothesis was that operative treatment would significantly improve body image. Secondly, referring to a cross-sectional aspect of the research, we aimed to investigate if differences in body image exist in AIS females following implementation (group one-CBT scoliosis sample, CBTSS) or not (group two-control scoliosis sample, CSS) of CBT support. A group of healthy female adolescents (healthy female sample, HFS) will also be selected for comparative purposes. Thus, we also hypothesized that CBTSS will exhibit more satisfaction with body shape and accuracy in estimating the severity of body deformity following surgery. 

## 2. Material and Methods

### 2.1. Recruitment to the Study

**Figure 1:**
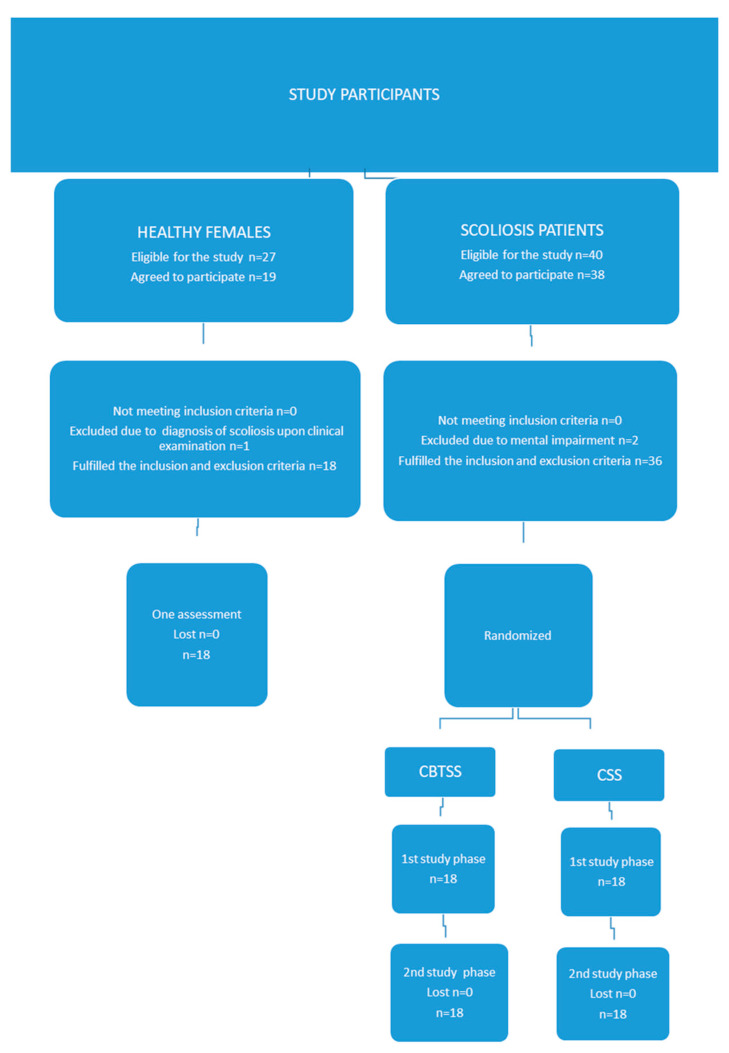
Participant flowchart.

Figure 1 provides a flowchart summarizing the participant recruitment process and data collection for the study. It outlines the stepwise enrollment procedure, indicating that 36 of the 40 eligible AIS patients participated in both the first and second phases of the study. In the HFS group, 29 participants were initially deemed eligible, with 10 declining participation and one excluded due to a postinvestigation diagnosis of AIS, resulting in a final sample of 18 participants. The participant recruitment period spanned from November 2022 to February 2024.

#### 2.1.1. Recruitment in the CBT Scoliosis Sample and Control Scoliosis Sample

The inclusion criteria were as follows: females aged 12–18 years; thoracic scoliosis; qualified for surgical treatment due to AIS using posterior spinal fusion. The exclusion criteria were previous spinal surgery, other severe medical conditions, and previous diagnosis of mental impairment. We decided to examine a group of females with thoracic scoliosis only. Earlier findings indicated that only the postoperative thoracic-apical translation significantly influenced the perception of trunk deformity. Specifically, a higher thoracic-apical translation reduced the likelihood of a positive perception of body shape after the scoliosis surgery [64].

Four versions of study information sheets were created to inform potential participants and their parents about the potential risks and benefits of participating in the trial: two for participants from the CBTSS and their parents, and two for participants from the CSS and their parents. These sheets were developed in collaboration with a psychologist and the study’s author (EM) prior to the initiation of the study.

Following the preparation of the information sheets, recruitment for the research commenced. A list of patients eligible for surgical treatment due to AIS, who met the entry criteria, was compiled. Using a random number table, individuals were randomly assigned to either the CBTSS (experimental group) or the CSS (control group) upon reporting to the surgical clinic. This method of simple randomization is effective in generating comparable sample sizes in both trial groups and in creating groups that are approximately equivalent with respect to both known and unknown prognostic factors. 

The recruitment occurred at the Department of Pediatric Orthopaedics and Traumatology and the Department of Spine Disorders and Pediatric Orthopedics, Poznan University of Medical Sciences, Poland. Patients were recruited to the study when they were admitted for scoliosis correction surgery at the beginning of their hospital stay. Two study authors (MG and MT) and a hospital psychologist contacted participating patients and their legal guardians. They were invited to participate in the study through a face-to-face conversation. In addition to the verbal information, the patients also received written information about their participation in the study. Both scoliosis samples (experimental and control) were fully informed of the study type. After that, they gave their informed written consent for participation in the study. Participants were provided with ongoing support throughout the hospital stay should they need explanations or clarifications about the survey.

To prevent contamination, participants in each group were instructed to discuss the intervention with everyone outside their group when the study concluded. No patients were mandated to return to the clinic for the research requirements. Participation was voluntary, and the patient and their parent could withdraw from the study without impacting their access to further treatment at the same clinic. The study protocol was thoroughly explained to all subjects during recruitment.

All patients were treated for idiopathic scoliosis by two orthopedic surgeons, the study’s authors (MG and MT). Patients had their scoliosis corrected with hybrid instrumentation using hooks and screws [65]. Scoliosis surgery was the initial spine surgery performed on these subjects. No postoperative complications that could have impacted the outcomes were identified.

The intervention module is outlined in Table S1 in the Supplementary Materials.

#### 2.1.2. Recruitment in the Healthy Female Sample

The following inclusion criteria for the HFS were adopted: females, age range of 12–18, and no scoliosis or other spinal deformities confirmed in the Adams test (clinical examination). The Adams forward bend test was used to assess suspected scoliosis in this sample, according to the methodology proposed by Santos [66].

Two versions of the study information sheets (for HFS participants and their parents) were developed to provide potential participants and parents with information about the possible benefits of participating in the trial. The schools attended by the students in the healthy female sample were chosen randomly, as were the class tutors to whom we sent a request containing information for students and their legal carers. They were fully informed of the study type. Then, they gave their informed written consent for participation in the study. 

During the Adams forward bend test, the pediatric orthopedist, a study author (MG), stood behind the students and instructed them to perform a trunk flexion, inclining the head and allowing the arms to drop towards the ground. The orthopedist assessed the symmetry of the thoracic and lumbar spines to detect any spinal deformity. Gibbosity, defined as an overcurvature opposed to contralateral flattening, was explicitly observed. The possible outcomes of this test were either suspicion of scoliosis (presence of gibbosity) or absence of scoliosis (absence of gibbosity) [66]. Following the procedure, each participant received written information from parents regarding the results of this examination. 

### 2.2. Ethical Issues

The study was conducted according to the guidelines of the Declaration of Helsinki. The study protocol was approved beforehand by the Bioethical Commission at the Poznan University of Medical Sciences (No. 695/18, No. 800/22) and by the Center for Safety Research at the University of Security in Poznan (No. 001/2018 and No. 001./2022).

### 2.3. The CBT Intervention in the CBT Scoliosis Sample

In our study, the hybrid CBT was implemented: participants received in-person therapy and, once home, had the option for ongoing support through telecommunication devices.

The in-person CBT intervention occurred before and after the surgery, during the AIS patients’ stay in the hospital ward. The number of planned therapeutic sessions depended on the patient’s needs. After discharge from the hospital, participants were provided continuity of CBT support for four months through telecommunication devices, such as e-mail, chat, and telephone, according to their preference. Patients could use those services, access CBT materials, or complete exercises. These online components included psychoeducational resources, such as articles detailing CBT strategies and teletherapy sessions with a therapist, which served as a supplement or alternative for in-person meetings.

Contacts were not scheduled and depended only on each patient’s needs, e.g., persistent negative automatic thoughts about patients’ bodies, dysfunctional assumptions about body shape, or body dissatisfaction. 

The duration of the intervention was tailored to maximize attendance while allowing sufficient time for participants to develop the necessary skills. An individual format was chosen based on evidence indicating no outcome difference between individual and group therapy [67]. The CBT was conducted by a CBT psychotherapist and modeled after Thompson’s CBT body image therapy [55]. Regarding the CBT protocol, Thompson’s CBT body image therapy is an example of a cognitive restructuring protocol specifically designed to address body image disturbances. Cognitive restructuring is a core component of this therapy and involves identifying, challenging, and modifying dysfunctional thoughts and beliefs about one’s body [55,68,69].

The content of CBT pre- and postsurgical sessions is outlined in Supplementary Materials.

### 2.4. Data Analyses

#### 2.4.1. Sociodemographic Data (CBT Scoliosis Sample, Control Scoliosis Sample, Healthy Female Sample)

We collected the following data from all study participants: current age, place of residence, school attended, weight, and height (to obtain body mass index, BMI).

#### 2.4.2. Radiological and Clinical Data (CBT Scoliosis Sample, Control Scoliosis Sample)

Radiological and clinical assessment was performed pre- and postsurgery. Standard X-Rays of the spine in anterior-posterior and lateral projections were performed. Parameters submitted for analysis included the Cobb angle of scoliosis in the main curve, the kyphosis angle in the thoracic spine, the lordosis angle in the lumbar spine, the distance (in centimeters) from the center of the vertebra at the scoliosis peak to the central sacral vertical line (CSVL), as a measurement of trunk decompensation and, defined as the degree of translation, scoliosis Lenke type, the location of the main curve, and percent of scoliosis correction after operative treatment.

Additionally, data on the age when scoliosis was diagnosed, data if scoliosis was previously diagnosed in a family member, and data on the duration of possible brace treatment (in months), as well as data on the presence of any chronic diseases, were gathered.

#### 2.4.3. Body Image (CBT Scoliosis Sample, Control Scoliosis Sample)

Adolescents’ body image was evaluated by means of a novel VR-based application called “Avatar Scoliosis 3D”. The detailed data concerning the assumptions, development, technical setup, and research procedures using this application was introduced in detail in our earlier study [51] and in Supplementary Materials.

**Figure 2:**
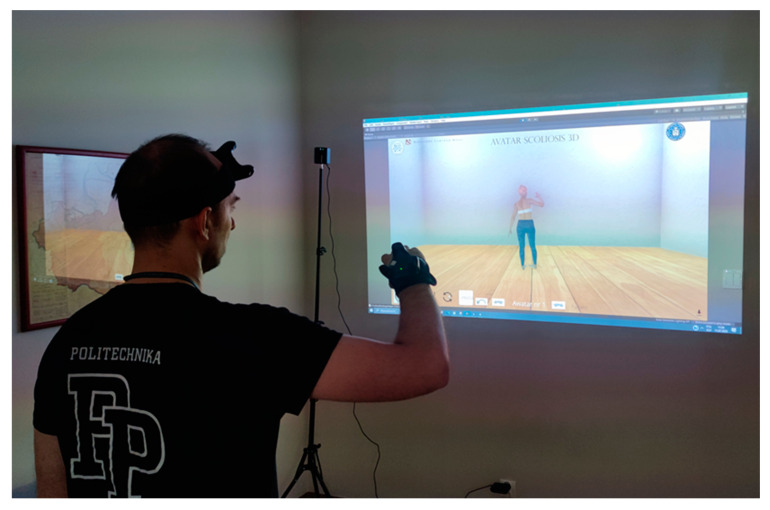
Internal testing of the “Avatar Scoliosis 3D” application.

The final version of the “Avatar Scoliosis 3D” application comprises hardware and software components. The hardware includes a projection device, specifically the Acer H6512BD projector (Acer Inc., New Taipei City, Taiwan), for large-screen projection of a 3D, movable avatar character. Motion data is captured using Vive Trackers 3.0 by the HTC company (HTC Corporation, New Taipei City, Taiwan). A total of six trackers are utilized and placed on the patient’s body: two on the hands, two on the feet, one on the forehead, and one on the waist (see Figure 2). 

Using this application, participants must choose those figures that best fit their self-perceived and desired body shape. The results of VR tasks were analyzed on an ordinal scale.

### 2.5. Statistical Methods

Regarding current age and age when scoliosis was diagnosed, weight, height, BMI, Cobb angle, kyphosis angle, lordosis angle, the degree of translation, percent of scoliosis correction, and duration of possible brace treatment (in months), the following indicators were determined: means, minimum and maximum values, and standard deviations (SDs). Concerning place of residence, school attended, scoliosis Lenke type, the location of the main curve, data if scoliosis was previously diagnosed in a family member, as well as data on the presence of any chronic diseases, the number of units that fell into the described categories of a given variable, along with their respective percentages, were determined. 

The results of the VR tasks were analyzed on an ordinal scale. Thus, from different experimental tasks conducted with the CBTSS, CSS, and HFS, the following indicators were extracted: (1st indicator) participants’ estimated current body shape at the time of E1; (2nd indicator) participants’ desired body shape at the time of E2; (3rd indicator) participants’ actual body shape (based on the radiographic parameter—the value of the Cobb angle—or clinical examination) (for the 1st, 2nd, and 3rd indicators, median, minimum, and maximum values and lower and upper quartiles were calculated); (4th indicator) the difference between the patients’ estimated current body shape at the time of E1, as compared to participants’ actual (based on the radiographic parameters—the value of the Cobb angle—or clinical examination) body shape; (5th indicator) difference between the desired body shape at the time of E2, as compared to participants’ actual (based on the radiographic parameters—the value of the Cobb angle—or clinical examination) body shape; (6th indicator) the difference between participants’ current estimated body shape during E1 and their desired body shape during E2.

Comparisons between two groups were performed using the t-test or the Mann–Whitney test. Comparisons between multiple groups were performed using the one-way ANOVA test, the F Welch test (with the Tukey multiple comparison test), or the Kruskal-Wallis test with the Dunn-Bonferroni multiple comparison tests. The accepted border level of statistical significance was 0.05; therefore, any test results exceeding this level were treated as insignificant. Statistical calculations were performed through Statistica 13.3 software (TIBCO Software Inc.)

## 3. Results

### 3.1. Characteristics of Study Samples

Table 1 presents the means, minimum and maximum values, and standard deviations (SDs), or the number of units that fell into the described categories of the given variable, along with their respective percentages, in relation to the sociodemographic, clinical, and radiological characteristics of study samples.

In the CBTSS, Lenke type 1 of scoliosis was identified in 12 patients (66.67), type 2 in 2 patients (11.11%), type 3 in 2 patients (11.11%), and type 6 in the remaining 2 patients (11.11%). Referring to the CSS, type 1 was indicated in 9 patients (50%), type 2 in 3 patients (16.67%), type 3 in 3 patients (16.67%), and type 6 in 3 patients (16.67%). 

Fifteen patients (83.33%) in CBTSS and 16 patients (88.89%) in CSS were prescribed bracing before surgery. 

The mean total duration of CBT in CBTSS was 11.61 hrs (SD 3.0, range 5–23). In this sample, two patients received 5 hrs of CBT, and one patient received 23 hrs of CBT (this one patient required support via teletherapy services). In the remaining 15 patients, the range of CBT duration was from 7 to 17 hrs. The additional data on all study groups are summarized in Table 2.

Considering the sociodemographic data, we indicated the difference between study groups in terms of place of residence (*p* = 0.00002) only. Considering clinical and radiological data, CBTSS and CSS differ significantly regarding preoperative and postoperative lordosis angle in the lumbar spine (*p* = 0.008 and *p* = 0.020, respectively).

### 3.2. VR Tasks

The median values for indicators 1, 2, and 3 are provided in Table 2. Regarding the 4th indicator, the analyzed differences are insignificant in both the CBTSS and CSS. This means that patients from both groups did not overestimate their perception of their current body shape as compared to their objective body shape. 

About the 5th indicator, the difference is significant preoperatively only, both in the CBTSS and CSS (*p* < 0.000001, the test power 0.99, and *p* < 0.000001, the test power 1.0, respectively). This means that patients from both scoliosis samples experience body dissatisfaction preoperatively but not postoperatively.

Regarding the last, 6th indicator, referring to the discrepancy between the estimated and desired body shape, differences are significant in CBTSS both pre- and postoperatively (*p* = 0.0007, the test power 0.99, and *p* = 0.029, the test power 0.96), respectively. In CSS, this difference is significant preoperatively (*p* = 0.002, the test power 1.0). The detailed results regarding VR tasks in the HFS are summarized in Table 2. 

### 3.3. Comparative Analyses

#### 3.3.1. Measurement of Body Image at Two Time Points

Table 3 presents the results of measurement of body image via VR tasks in the CBTSS and CSS at two time points. The comparative analyses were planned as follows: presurgical/postsurgical.

Regarding VR tasks, the only significant differences between pre- and postsurgical results were confirmed, as expected for the 1st and 3rd indicators in both the CBTSS and CSS (*p* = 0.002, the test power 1.0, and *p* = 0.002, the test power 1.0, respectively). For details, see Table 3. 

#### 3.3.2. Cross-Sectional Analyses

Table 4 presents the results of cross-sectional analyses referring to VR tasks between the CBTSS, CSS, and HFS. Comparisons between the two groups were performed using the t-test or the Mann–Whitney test. Comparisons between multiple groups were performed using the one-way ANOVA test, the F Welch test (with the Tukey multiple comparison test), or the Kruskal–Wallis test with the Dunn-Bonferroni multiple comparison tests.

Regarding the VR tasks (1st to 3rd indicators), significant differences occurred preoperatively only (*p* < 0.00001, the test power 1.0, *p* < 0.000001, the test power 0.61, *p* < 0.000001, the test power 1.0, respectively). 

The results of post-hoc tests revealed differences, as expected, between CBTSS and HHS and CSS and HFS but not between CBTSS and CSS.

Any significant differences between all study groups were identified in relation to postsurgical comparisons.

## 4. Discussion

In this study, we examined if operative treatment due to AIS would significantly improve body image in adolescent females. We found that, generally, AIS patients experienced dissatisfaction with body shape preoperatively but not postoperatively. 

Our objective was also to determine if differences in body image exist in AIS females following the implementation of CBT support. We revealed that irrespective of received therapeutic support, AIS patients accurately estimate their body shape pre- and postoperatively, as well, as they feel dissatisfied with their body preoperatively but not postoperatively.

The study utilized an innovative VR-based methodology to preliminarily test the proposed hypotheses, representing a unique contribution to body image assessment in AIS. The use of VR in scoliosis assessment offers several advantages. Firstly, VR introduces a third dimension to the test silhouettes, enhancing assessment accuracy [46,47]. Specifically, it enables participants to discriminate stimuli better, leading to more precise responses and estimations [47]. The 3D visuals allow participants to effectively perceive scoliosis-related deformities, such as shoulder asymmetry, rib humps, or waistline asymmetry.

Secondly, the immersive nature of VR enables users to interact with a virtual figure within a shared virtual environment, ensuring that the figure is displayed at a life-like scale [50]. This realistic full-body avatar responds to real-time user movements across all planes of motion [51]. The ‘Avatar Scoliosis 3D’ application allows patients to view their virtual figures in a manner that closely resembles their actual physical shape. By using VR glasses or head-mounted displays, patients can confront their virtual bodies in real time, facilitating a more robust identification with their postsurgical appearance. This, in turn, may contribute to greater satisfaction with the aesthetic outcomes of surgery. 

About the part of the study that examined the importance of surgical treatment with body image, several studies have explored the impact of AIS on patients’ body representation. They mainly involved longitudinal research, with pre- and postsurgical evaluations, to assess changes over time in response to treatments such as bracing or surgery. The most significant evidence for improved body image among AIS patients comes from surgical intervention. 

As stated above, we found that, generally, AIS patients experienced dissatisfaction with body shape preoperatively but not postoperatively. It is worth noting that numerous studies consistently report that patients tend to express remarkably high levels of satisfaction following scoliosis surgery. Bago et al. [70] reported that 90% of patients were pleased with their surgical results, with 87% stating they would choose the same intervention again. Furthermore, 91% of patients reported feeling better or significantly better postoperatively compared to their preoperative state. Albayrak et al. [71] similarly observed enhanced satisfaction among adolescents after scoliosis surgery, as reflected in improved SRS-22 scores after comparing preoperative and postoperative full-spine images. These findings are consistent with the results we obtained. 

However, Noonan et al. have shown that a diminished body image may persist for many years in AIS patients following surgery [72], which contradicts our conclusions. It would be interesting to investigate further, using VR, which psychological variables are associated with dissatisfaction following scoliosis surgery. Sieberg et al. [73], for instance, explored how presurgical mental health, pain, and self-image in adolescents with idiopathic scoliosis affected their satisfaction two years after spinal fusion surgery. They also examined the role of presurgical expectations regarding spinal appearance, yet surprisingly, presurgical self-image did not correlate with postsurgical satisfaction [74].

Given the importance of psychological interventions for AIS patients undergoing spinal surgery, in the current study, we focused on only one intervention category (CBT). It is inspired by the CBT body image therapy of Thompson [55]. Utilizing modern VR techniques, we focused on two body image issues: distortion and dissatisfaction. 

In the context of other psychological interventions for patients undergoing spinal surgery, a systematic review conducted by van Niekerk et al. [74] identified six studies specifically addressing patients with AIS undergoing spinal surgery [74,75,76,77,78,79,80]. The majority of these studies were randomized clinical trials (RCTs) [76,77,78,79,80,81,82] and systematically excluded participants with psychological, cognitive, and developmental conditions [75,76,77,78,81,82]. The review highlighted two primary categories of interventions: brief educational programs designed to help patients manage pain and anxiety postsurgery [76,77,78,80,81] and intensive multidisciplinary care models [75,82]. For instance, Ying and Fu [82] compared routine nursing care and Rosenthal effect-based nursing. The latter involved proactive mental healthcare provided by nurses following spinal surgery, along with training for family members to monitor the patient’s mental well-being. The authors found that this intervention significantly outperformed routine medical care by reducing depression and anxiety levels, enhancing quality of life, decreasing pain intensity, and improving patient satisfaction with nursing care [82].

In the context of our study, and contrary to our expectations, we revealed that patients from both scoliosis samples, with and without CBT intervention, accurately estimated their body shape pre- and postoperatively. Thus, they did not suffer from body image distortion. Furthermore, both CBTSS and CSS, irrespective of received therapeutic support, were satisfied with the cosmetic result of scoliosis surgery. It is worth verifying if this result is stable in a longer time frame, not only immediately after the hospital ward’s spinal surgery. 

Our results are consistent with the findings of Hinrichsen et al. [83], who conducted a cross-sectional study comparing the mental well-being of AIS patients attending a scoliosis self-help group with those who had sought information about the group sessions but had yet to participate. Their study revealed no significant differences between the groups in most outcomes, including psychosomatic symptoms [83]. 

The remaining results, inconsistent with the presented study hypothesis, also warrant further discussion. As pointed out above, AIS patients’ dissatisfaction with body shape was present preoperatively, as expected, both in the CBTSS and CSS. However, this discrepancy remained, surprisingly, significant immediately following the surgery in the CBTSS only. This tendency could be verified. However, referring to another factor of patient dissatisfaction (5th indicator, disparities between the desired and actual body shape), it was revealed that patients from both scoliosis samples experienced body dissatisfaction preoperatively but not postoperatively. This inconsistency in study results may have occurred due to the relatively small sample sizes. Furthermore, as indicated above, due to the specific technical setup of the “Scoliosis 3D” application, we could not perform VR tasks at patients’ homes and verify if this tendency remained stable in a longer follow-up after the surgery. This issue should be checked during a planned hospital check-up and after continuing CBT sessions.

## 5. Conclusions

Considering the patients’ accuracy in the estimation of the severity of body deformity (4th indicator in VR tasks analysis), we revealed in both clinical samples that patients did not over- or underestimate their perception of current body shape as compared to their objective (based on radiographic parameters) body shape, which is contrary to our expectations. This result also might have occurred due to the relatively small sample sizes. In the clinical context, this means that AIS patients might not need support in learning a more accurate perception of the severity of their deformities. It would be interesting to verify if this trend is similar during AIS brace treatment in females with less severe trunk deformities. At the same time, future studies would benefit from investigating if those results are stable in the long-term follow-up. 

Some limitations of the present study should be noted. Firstly, a notable limitation is that the VR assessment had not yet been validated prior to the study, and no additional body-image measurements were utilized to establish the convergent validity of this novel assessment method. This may impact the significance of the study results. However, a pilot testing of “Avatar Scoliosis 3D” psychometric properties: validity and test-retest reliability yielded acceptable results. At the same time, the current study did not address, e.g., floor and ceiling effects or construct validity of “Avatar Scoliosis 3D”. Similarly, other psychological variables influencing body image, e.g., emotional awareness, emotion regulation, or level of anxiety due to physical appearance, have not been studied either. Future research should include a more detailed examination of “Avatar Scoliosis 3D” psychometric properties.

Secondly, the clinical sample size at baseline (n = 36 in total) was relatively small, which could limit the generalizability of the study results. Moreover, drop-outs from the final follow-up evaluation must be noted, especially in the CSS. A smaller sample would give us results that may need to be sufficiently powered to detect a difference between the CBTSS and CSS, and the study may turn out to be falsely negative, leading to a type II error. However, the test power was calculated. While the small sample size may have contributed to the lack of significant results, it is also possible that the specific CBT protocol used in this study was not optimally tailored to address the unique needs of this patient population.

Additionally, the duration of the intervention may have needed to be increased to produce measurable changes in the outcomes assessed. It must be noted that it was a relatively brief intervention suitable for patients with moderate body image concerns. The length of Thompson’s CBT body image therapy, like many CBT protocols, can vary depending on several factors, including the severity of the body image disturbance or the individual client’s progress. In our study, additional factors were related to a relatively short hospital stay before the surgery and the psychophysical condition of a patient after a major spine surgery.

Thirdly, the current study involved female patients only. This inclusion criterion may have impacted the distribution of scores and, in that way, limited the generalizability of the findings on the whole population of AIS patients. In addition, our VR-based methodology was designed to investigate body image in thoracic scoliosis patients only. Thus, in another study, a similar VR-based method could be developed for lumbar or thoracolumbar male and female scoliosis patients to investigate specific locations of deformities, e.g., waist asymmetry, and their consequences for body image dissatisfaction or distortion. Regarding gender differences, societal norms and expectations regarding body image may differ, affecting how each gender experiences and addresses scoliosis. Considering these factors will enhance the depth and relevance of future research. 

Referring to CBT was not standardized across patients within the CBTSS. This factor could have an impact on the study results. For example, any differences between studied samples might be due to variables outside of the intervention, e.g., patients’ engagement or total duration of CBT intervention. We are aware that a typical course of Thompson’s CBT body image therapy usually ranges from 7 to 14 sessions. On the other hand, therapists often adopt a flexible approach, adjusting the length and intensity of therapy based on ongoing assessments of the client’s needs and progress. Regular feedback and open communication between the therapist and client are crucial to ensuring the therapy remains compelling and appropriately paced. A more extended intervention would allow for a more in-depth exploration of cognitive distortions, more time for practicing and reinforcing new cognitive and behavioral skills, and the development of robust relapse prevention strategies. Such an intervention could affect the results of the present study. 

Moreover, the current study has many strengths that justify its value. Due to a novel methodology, our research was a pioneering study in a new field, and it was challenging to gather a larger sample. In addition, the study sample was very homogeneous, with strict inclusion and exclusion criteria. Furthermore, nonparametric statistical tests were applied to verify study hypotheses. The results of the study can greatly influence practical applications in the field. Lastly, the study’s findings provide a basis for further, more extensive studies that can confirm and expand upon the conclusions.

In light of the implications of the current study for future research, several recommendations have already been noted. An additional recommendation is to evaluate the efficacy of CBT for AIS over an extended duration to determine whether the beneficial effects of this therapeutic intervention on body representation and body experience are maintained. Furthermore, future investigations would benefit from examining other psychological variables that may have affected the study outcomes and incorporating these variables at baseline in longitudinal analyses. The application “Avatar Scoliosis 3D” could also be a valuable tool for assessing body image distortion in AIS patients during conservative treatment.

In summary, the innovative aspect of this research lies in evaluating CBT interventions using a novel VR-based methodology. Additionally, the inclusion of a control group of healthy female adolescents provided valuable insights into the phenomena of body deformity overestimation and body dissatisfaction in AIS. Our findings indicate that AIS patients accurately assess their body shape preoperatively and postoperatively, regardless of the therapeutic support received. However, they experience body dissatisfaction preoperatively that does not persist postoperatively. Consequently, it is recommended that CBT interventions be individually tailored to address these issues effectively.

## Figures and Tables

**Table 1:** Sociodemographic, clinical, and radiological characteristics of study samples.

	CBTSS			CSS			HFS			*p* Comparison CBTSS/CSS/HFS
	Mean (SD)	Range (Min–Max)	n (%)	Mean (SD)	Range (Min–Max)	n (%)	Mean (SD)	Range (Min–Max)	n (%)	
Sociodemographic characteristics										
Place of residence										0.00002 *
Rural	*p* = 0.060	-	3 (16.67)	-	-	7 (38.89)	-	-	0 (0)	
City below 25,000 inhabitants	*p* = 0.207	-	8 (44.4)	-	-	2 (11.11)	-	-	3 (16.67)	
A city between 25,000 and 200,000 inhabitants	-	-	4 (22.22)	-	-	7 (38.89)	-	-	1 (5.56)	
City with over 200,000 inhabitants	-	-	3 (16.67)	-	-	2 (11.11)	-	-	14 (77.77)	
School Attended	-									
Elementary	-	-	9 (50.00)	-	-	9 (50.00)	-	-	15 (83.33)	0.060
Secondary	-	-	9 (50.00)	-	-	9 (50.00)	-	-	3 (16.66)	
Age at current [years]	14.17 (2.01)	12–17	-	14.50 (1.50)	12–17	-	13.56 (1.15)	12–16	-	0.060
Age at scoliosis diagnosis [years]	9.9 (3.10)	5–14	-	10.11 (2.56)	5–14	-	-	-	-	0.816
Weight [kg]	53.22 (9.61)	35–70	-	52.33 (11.01)	38–85	-	62.00 (15.71)	40–100	-	0.086
Height [cm]	160.17 (6.40)	149–174	-	160.89 (5.99)	153–172	-	163.00 (6.75)	153–176	-	0.392
Body Mass Index	20.63884 (2.88)	15.77–27.01	-	20.16 (3.8)	16.14–31.60	-	23.19 (5.06)	17.09–35.42	-	0.076
Family history of scoliosis	-	-	5 (27.78)	-	-	6 (33.33)	-	-	2 (11.11)	0.370
Comorbidities	-	-	3 (16.67)	-	-	3 (16.67)	-	-	4 (22.22)	<1
Clinical and radiological characteristics	CBTSS				CSS				*p* comparison CBTSS/CSS (preoperatively/ postoperatively)	
	Preoperatively		Postoperatively		Preoperatively		Postoperatively			
	Mean (SD)	Range (Min-Max)	Mean (SD)	Range (Min-Max)	Mean (SD)	Range (Min-Max)	Mean (SD)	Range (Min-Max)		
Duration of CBT [hours]	5.22 (2.0)	2–9	5.80 (2.24)	1–9	-					
Brace treatment [in months]	24.11 (20.20)	0–72	-		35.06 (25.43)	0–84	-		0.162	
Cobb angle in the main curve	61.33 (8.0)	52–78	23.22 (7.69)	16–50	62.16 (10.70)	46–86	24.27 (8.76)	12–46	0.793/0.466	
Thoracic kyphosis angle	26.33 (19.11)	6–40	18.94 (7.99)	7–37	21.22 (11.31)	4–38	18.28 (7.58)	4–32	0.419/0.799	
Lumbar lordosis angle	48.61 (9.98)	24–70	39.28 (10.44)	12–64	38.33 (11.72)	15–60	30.59 (10.95)	6–55	0.008 */0.020 *	
Apical translation [cm]	4.43 (1.96)	0.3–8.7	1.51 (1.31)	0–5.8	4.97 (1.87)	1.3–8.6	1.68 (0.82)	0.5–3.2	0.410/0.254	
% of scoliosis correction	-		60.50 (11.67)	34–76	-		61.50 (11.46)	38–81	0.949	

Note. CBT SS-CBT scoliosis sample; CSS—control scoliosis sample; HFS—healthy female sample; * *p* < 0.05.

**Table 2:** The virtual reality tasks descriptive statistics.

VR Tasks	Median	Range (Minimum–Maximum)	Lower Quartile–Upper Quartile	Median	Range (Minimum–Maximum)	Lower Quartile–Upper Quartile	Median	Range (Minimum–Maximum)	Lower Quartile–Upper Quartile
	CBTSS Preoperatively/Postoperatively			CSS Preoperatively/Postoperatively			HFS		
1st indicator	4.00/2.00	3.00–7.00/ 1.00–3.00	3.00–6.00/ 2.00–3.00	4.00/2.00	2.00–7.00/ 1.00–4.00	3.00–5.00/ 2.00/2.00	1.00	1.00–4.00	1.00–2.00
2nd indicator	2.00/1.00	1.00–3.00/ 1.00–3.00	1.00–3.00/ 2.00–3.00	2.00/2.00	1.00–3.00/ 1.00–3.00	1.00–2.00/ 1.00/2.00	1.00	1.00–3.00	1.00–1.00
3rd indicator	6.00/2.00	5.00–7.00/ 1.00–5.00	5.00–6.00/ 1.00–2.00	6.00/2.00	4.00–7.00/ 1.00–4.00	5.00–7.00/ 1.00–2.00	1.00	1.00–1.00	1.00–1.00
*p*									
4th indicator	0.287/0.908			0.907/1			0.090		
5th indicator	<0.000001 */1			<0.000001 */1			1		
6th indicator	0.0007 */0.029 *			0.002 */0.287			0.200		

Note. 1st indicator: participants’ estimated current body shape at the time of E1; 2nd indicator: participants’ desired body shape at the time of E2; 3rd indicator: participants’ actual body shape (based on the radiographic parameters or clinical examination); 4th indicator: difference between the patients’ estimated current body shape at the time of E1, as compared to participants’ actual (based on the radiographic parameters or clinical examination) body shape; 5th indicator: the difference between the desired body shape at the time of E2, as compared to participants’ actual (based on the radiographic parameters or clinical examination) body shape; 6th indicator: the difference between participants’ current estimated body shape at the time of E1, after comparison to participants’ desired body shape at the time of E2; CBT SS-CBT scoliosis sample; CSS—control scoliosis sample; HFS—healthy female sample; VR—virtual reality; * *p* < 0.05.

**Table 3:** Measurement of body image at two time points in CBTSS and CSS.

VR Tasks	*p* Comparison Presurgical-Postsurgical	
1st indicator	0002 *	0.0002 *
2nd indicator	0.142	0.361
3rd indicator	0.0002 *	0.0002 *

Note. 1st indicator: participants’ estimated current body shape at the time of E1; 2nd indicator: participants’ desired body shape at the time of E2; 3rd indicator: participants’ actual body shape (based on the radiographic parameters or clinical examination; CBT SS-CBT scoliosis sample; CSS—control scoliosis sample; VR—virtual reality; * *p* < 0.05.

**Table 4:** Cross-sectional analyses between the CBTSS, CSS, and HFS.

	Preoperatively	Postoperatively
	*p* Comparison CBTSS/CSS/HFS	*p* Comparison CBTSS/CSS/HFS
VR tasks		
1st indicator	<0.00001 *, CA: 1, CB: 0.0009 *, CC: 0.00008 *	0.314
2nd indicator	<0.000001 *, CA: 1, CB: 0.000004 *, CC = 0.000007 *	0.312
3rd indicator	<0.000001 *, CA: 1, CB: 0.000002 *, CC: 0.0000001 *	0.850

Note. CBT SS-CBT scoliosis sample; CSS—control scoliosis sample; HFS—healthy female sample; 1st indicator: participants’ estimated current body shape at the time of E1; 2nd indicator: participants’ desired body shape at the time of E2; 3rd indicator: participants’ actual body shape (based on the radiographic parameters or clinical examination); CA—Comparison A: CBTSS/CSS, CB—Comparison B: CBTSS/HFS; CC—Comparison C: CSS/HFS; * *p* < 0.05.

## Data Availability

The data presented in this study are available upon request from the corresponding author. The data are not publicly available due to privacy.

## Supplementary Materials

The following supporting information can be downloaded at: https://www.mdpi.com/article/10.3390/jcm13216422/s1. Table S1: Intervention module; Table S2: Preliminary psychometric properties of “Avatar Scoliosis 3D”.

## References

[1] MacLean W.E., Green N.E., Pierre C.B., Ray D.C. (1989). Stress and coping with scoliosis: Psychological effects on adolescents and their families. J. Pediatr. Orthop..

[2] Matsunaga S., Hayashi K., Naruo T., Nozoe S., Komiya S. (2005). Psychologic management of brace therapy for patients with idiopathic scoliosis. Spine.

[3] Danielsson A.J., Wiklund I., Pehrsson K., Nachemson A.L. (2001). Health-related quality of life in patients with adolescent idiopathic scoliosis: A matched follow-up at least 20 years after treatment with brace or surgery. Eur. Spine J..

[4] Cash T.F., Sarwer D.B., Pruzinsky T., Cash T.F., Goldwyn R.M., Persing J.A., Whitaker L.A. (2006). Body image and plastic surgery. Psychological Aspects of Reconstructive and Cosmetic Plastic Surgery: Clinical, Empirical and Ethical Perspectives.

[5] Auerbach J.D., Lonner B.S., Crerand C.E., Shah S.A., Flynn J.M., Bastrom T., Penn P., Ahn J., Toombs C., Bharucha N. (2014). Body image in patients with adolescent idiopathic scoliosis: Validation of the Body Image Disturbance Questionnaire—Scoliosis Version. J. Bone Jt. Surg. Am..

[6] Weinstein S.L. (1994). Adolescent idiopathic scoliosis: Prevalence and nature history. The Pediatric Spine: Principle and Practice.

[7] Chung N., Cheng Y.H., Po H.L., Ng W.K., Cheung K.C., Yung H.Y., Lai Y.M. (2018). Spinal phantom comparability study of Cobb angle measurement of scoliosis using digital radiographic imaging. J. Orthop. Translat..

[8] Lehmann T.P., Juzwa W., Filipiak K., Sujka-Kordowska P., Zabel M., Głowacki J., Głowacki M., Jagodziński P.P. (2016). Quantification of the asymmetric migration of the lipophilic dyes, DiO and DiD, in homotypic co-cultures of chondrosarcoma SW-1353 cells. Mol. Med. Rep..

[9] Glowacki M., Ignys-O’Byrne A., Ignys I., Wroblewska K. (2011). Limb shortening in the course of solitary bone cyst treatment—A comparative study. Skeletal Radiol..

[10] Wang J., Zhang J., Xu R., Chen T.G., Zhou K.S., Zhang H.H. (2018). Measurement of scoliosis Cobb angle by end vertebra tilt angle method. J. Orthop. Surg. Res..

[11] Głowacki M. (2002). Wartość Wybranych Czynników Prognostycznych w Leczeniu Operacyjnym Skoliozy Idiopatycznej.

[12] Wong A.Y.L., Samartzis D., Cheung P.W.H., Cheung J.P.Y. (2019). How Common Is Back Pain and What Biopsychosocial Factors Are Associated with Back Pain in Patients with Adolescent Idiopathic Scoliosis?. Clin. Orthop. Relat. Res..

[13] Clayson D., Luz-Alterman S., Cataletto M.M., Levine D.B. (1987). Longterm psychological sequelae of surgically versus nonsurgically treated scoliosis. Spine.

[14] Misterska E., Glowacki M., Harasymczuk J. (2010). Personality characteristics of females with adolescent idiopathic scoliosis after brace or surgical treatment compared to healthy controls. Med. Sci. Monit..

[15] Ashman R.B., Herring J.A., Johnston C.E., Ashman R.B., Herring J.A., Johnston C. (1993). E & Hundley and Associates. TSRH Spinal Instrumentation.

[16] Tolo V.T. (1991). Surgical treatment of adolescent idiopathic scoliosis. Semin. Spine Surg. Scoliosis.

[17] Haher T.R., Merola A., Zipnick R.I., Gorup J., Mannor D., Orchowski J. (1995). Meta-analysis of surgical outcome in adolescent idiopathic scoliosis: A 35-year English literature review of 11,000 patients. Spine.

[18] Theologis T.N., Jefferson R.J., Simpson AH R.W., Turner-Smith A.R., Fairbank J.C.T. (1993). Quantifying the cosmetics defect of adolescent idiopathic scoliosis. Spine.

[19] Sattin D., Parma C., Lunetta C., Zulueta A., Lanzone J., Giani L., Vassallo M., Picozzi M., Parati E.A. (2023). An Overview of the Body Schema and Body Image: Theoretical Models, Methodological Settings and Pitfalls for Rehabilitation of Persons with Neurological Disorders. Brain Sci..

[20] Bertuccelli M., Cantele F., Masiero S. (2023). Body Image and Body Schema in Adolescents with Idiopathic Scoliosis: A Scoping Review. Adolescent. Res. Rev..

[21] Cantele F., Maghini I., Tonellato M., Meneguzzo P., Favaro A., Masiero S. (2020). An analysis of eating disorders in adolescent idiopathic scoliosis. Spine.

[22] Yagci G., Karatel M., Yakut Y. (2020). Body awareness and its relation to quality of life in individuals with idiopathic scoliosis. Percept. Mot. Skills.

[23] Brewer P., Berryman F., Baker D., Pynsent P., Gardner A. (2013). Influence of Cobb angle and ISIS2 surface topography volumetric asymmetry on scoliosis research society-22 outcome scores in scoliosis. Spine Deform..

[24] Watanabe K., Hasegawa K., Hirano T., Uchiyama S., Endo N. (2007). Evaluation of postoperative residual spinal deformity and patient outcome in idiopathic scoliosis patients in Japan using the scoliosis research society outcomes instrument. Spine.

[25] Duramaz A., Yılmaz S., Ziroğlu N., Bursal Duramaz B., Kara T. (2018). The effect of deformity correction on psychiatric condition of the adolescent with adolescent idiopathic scoliosis. Eur. Spine J..

[26] Lonner B.S., Brochin R., Lewis R., Vig K.S., Kassin G., Castillo A., Ren Y. (2019). Body image disturbance improvement after operative correction of adolescent idiopathic scoliosis. Spine Deform..

[27] Farrell C., Lee M., Shafran R. (2005). Assessment of body size estimation: A review. Eur. Eat. Disord. Rev..

[28] Gardner R.M., Brown D.L. (2014). Body size estimation in anorexia nervosa: A brief review of findings from 2003 through 2013. Psychiatry Res..

[29] Cash T.F., Deagle E.A. (1997). The nature and extent of body-image disturbances in anorexia nervosa and bulimia nervosa: A meta-analysis. Int. J. Eat. Disord..

[30] Mölbert S.C., Thaler A., Mohler B.J., Streuber S., Romero J., Black M.J., Zipfel S., Karnath H.O., Giel K.E. (2017). Assessing body image in anorexia nervosa using biometric self-avatars in virtual reality: Attitudinal components rather than visual body size estimation are distorted. Psychol. Med..

[31] Manzoni G.M., Cesa G.L., Bacchetta M., Castelnuovo G., Conti S., Gaggioli A., Mantovani F., Molinari E., Cárdenas-López G., Riva G. (2016). Virtual Reality-Enhanced Cognitive-Behavioral Therapy for Morbid Obesity: A Randomized Controlled Study with 1 Year Follow-Up. Cyberpsychol Behav. Soc. Netw..

[32] Roy M. (2005). Quantification de la Distorsion de l’image Corporelle chez des Adolescentes Atteintes d’anorexie Mentale Restrictive: Évaluation Informatique (Q-DIC) et Applications Cliniques. Master’s Thesis.

[33] Collins J.K. (1986). The objective measurement of body image using video technique: Reliability and validity studies. Br. J. Psychol..

[34] Freeman R.J., Thomas C.D., Solyom L., Hunter M. (1984). A modified video-camera for measuring body image distortion: Technical description and reliability. Psychol. Med..

[35] Gardner R.M., Stark K., Jackson N.A., Friedman B.N. (1999). Development and validation of 2 new scales for assessment of body image. Percept. Mot. Skills.

[36] Thompson M.A., Gray J.J. (1995). Development and validation of a new body image assessment tool. J. Pers. Assess..

[37] Doll M., Ball G.D.C., Willows N.D. (2004). Rating of figures used for body image assessment varies depending on the method of figure presentation. Int. J. Eat. Disord..

[38] Lewis J.S., McCabe C.S. (2010). Body Perception Disturbance (BPD) in CRPS. Pract. Pain. Manag..

[39] Mehling W.E., Gopisetty V., Daubenmier J., Price C.J., Hecht F.M., Stewart A. (2009). Body Awareness: Construct and Self-Report Measures. PLoS ONE.

[40] Moseley G.L. (2008). I can’t find it!. Distorted body image and tactile dysfunction in patients with chronic back pain. Pain.

[41] Roosink M., McFadyen B.J., Hébert L.J., Jackson P.L., Bouyer L.J., Mercier C. (2015). Assessing the perception of trunk movements in military personnel with chronic non-specific low back pain using a virtual mirror. PLoS ONE.

[42] Hodges P.W., Smeets R.J. (2014). Interaction between pain, movement, and physical activity: Short-term benefits, long-term consequences, and targets for treatment. Clin. J. Pain..

[43] Ferrer-García M., Gutiérrez-Maldonado J. (2012). The use of virtual reality in the study, assessment, and treatment of body image in eating disorders and nonclinical samples: A review of the literature. Body Image.

[44] Milgram P., Takemura H., Utsumi A., Kishino F. (1995). Augmented reality: A class of displays on the reality-virtuality continuum. Telemanipulator Telepresence Technol..

[45] Skarbez R., Smith M., Whitton M.C. (2021). Revisiting Milgram and Kishino’s reality-virtuality continuum. Front Virtual Real..

[46] Riva G., Malighetti C., Serino S. (2021). Virtual reality in the treatment of eating disorders. Clin. Psychol. Psychother..

[47] Riva G. (2022). Virtual Reality in Clinical Psychology. Compr. Clin. Psychol..

[48] Perpiñá C., Botella C., Baños R., Marco H., Alcañiz M., Quero S., Peñarrocha V. (2023). The use of virtual reality in the study, assessment, and treatment of body image in eating disorders and nonclinical samples. Behav. Modif..

[49] Perpiñá C., Botella C., Baños R.M. (2000). Imagen Corporal en los Trastornos Alimentarios. Evaluación y Tratamiento Mediante Realidad Virtual.

[50] Gorini A., Capideville C.S., De Leo G., Mantovani F., Riva G. (2011). The role of immersion and narrative in mediated presence: The virtual hospital experience. Cyberpsychol Behav. Soc. Netw..

[51] Misterska E., Górski F., Tomaszewski M., Bun P., Gapsa J., Słysz A., Głowacki M. (2023). “Scoliosis 3D”—A Virtual-Reality-Based Methodology Aiming to Examine AIS Females’ Body Image. Appl. Sci..

[52] Koch K.D., Buchanan R., Birch J.G., Morton A.A., Gatchel R.J., Browne R.H. (2001). Adolescents undergoing surgery for idiopathic scoliosis: How physical and psychological characteristics relate to patient satisfaction with the cosmetic result. Spine.

[53] Koemeda-Lutz M., Kaschke M., Revenstorf D., Scherrmann T., Weiss H., Soeder U. (2006). Evaluation of the effectiveness of body-psychotherapy in out-patient settings (EEBP). Psychother. Psychosom. Med. Psychol..

[54] Rohricht F., Papadopoulos N., Priebe S. (2013). An exploratory randomized controlled trial of body psychotherapy for patients with chronic depression. J. Affect. Disord..

[55] Thompson J.K. (1996). Body Image, Eating Disorders, and Obesity.

[56] Grant J.R., Cash T.F. (1995). Cognitive-behavioral body-image therapy: Comparative efficacy of group and modest-contact treatments. Behav. Ther..

[57] Rosen J.C., Saltzberg E., Srebnik D. (1989). Cognitive behavior therapy for negative body image. Behav. Ther..

[58] Butters J.W., Cash T.F. (1987). Cognitive-behavioral treatment of women’s body-image dissatisfaction. J. Consult. Clin. Psychol..

[59] Jarry J.L., Ip K. (2005). The effectiveness of stand-alone cognitive-behavioural therapy for body image: A meta-analysis. Body Image.

[60] Veale D. (2004). Advances in a cognitive behavioural model of body dysmorphic disorder. Body Image.

[61] Rosen J.C., Reiter J., Orosan P. (1995). Cognitive-behavioral body image therapy for body dysmorphic disorder. J. Consult. Clin. Psychol..

[62] Khemlani-Patel S., Neziroglu F., Mancusi L.M. (2011). Cognitive-behavioral therapy for body dysmorphic disorder: A comparative investigation. Int. J. Cogn. Ther..

[63] Allen A. (2006). Cognitive-behavioral treatment of body dysmorphic disorder. Prim. Psychiatry.

[64] Misterska E., Głowacki M., Harasymczuk J. (2011). Assessment of spinal appearance in female patients with adolescent idiopathic scoliosis treated operatively based on Spinal Appearance Questionnaire. Med. Sci. Monit..

[65] Kim Y.J., Lenke L.G., Kim J., Bridwell K.H., Cho S.K., Cheh G., Sides B. (2006). Comparative analysis of pedicle screw versus hybrid instrumentation in posterior spinal fusion of adolescent idiopathic scoliosis. Spine.

[66] Santos C.I., Cunha A.B., Braga V.P., Saad I.A., Ribeiro M.A., Conti P.B. (2009). Occurrence of postural deviations in children of a school of jaguariúna. Rev. Paul. Pediatr..

[67] Turner-Stokes L., Erkeller-Yuksel F., Miles A., Pincus T., Shipley M., Pearce S. (2003). **Outpatient cognitive behavioral** pain management programs: A randomized comparison of a group-based multidisciplinary versus an individual therapy model. Arch. Phys. Med. Rehabil..

[68] Cash T.F. (2008). The Body Image Workbook: An Eight-Step Program for Learning to Like Your Looks.

[69] Thompson J.K., Heinberg L.J., Altabe M., Tantleff-Dunn S. (1999). Exacting Beauty: Theory, Assessment, and Treatment of Body Image Disturbance.

[70] Bago J., Perez-Grueso F., Les E., Hernandez P., Pellise F., Fernandez-Baillo N., Villanueva C., Garcia A. (2009). Clinical outcome of surgery for idiopathic scoliosis. Evaluation by satisfaction with treatment and overall perceived effect. Eur. Spine J..

[71] Albayrak A., Buyuk A.F., Ucpunar H., Balioglu M.B., Kargin D., Kaygusuz M.A. (2015). Pre- and postoperative photographs and surgical outcomes in patients with Lenke type 1 adolescent idiopathic scoliosis. Spine.

[72] Noonan K.J., Dolan L.A., Jacobson W.C., Weinstein S.L. (1997). Long-term psychosocial characteristics of patients treated for idiopathic scoliosis. J. Pediatr. Orthop..

[73] Sieberg C.B., Manganella J., Manalo G., Simons L.E., Hresko M.T. (2017). Predicting Postsurgical Satisfaction in Adolescents With Idiopathic Scoliosis: The Role of Presurgical Functioning and Expectations. J. Pediatr. Orthop..

[74] van Niekerk M., Richey A., Vorhies J., Wong C., Tileston K. (2023). Effectiveness of psychosocial interventions for pediatric patients with scoliosis: A systematic review. World J. Pediatr. Surg..

[75] Chan C.Y.W., Loo S.F., Ong J.Y., Lisitha K.A., Hasan M.S., Lee C.K., Chiu C.K., Kwan M.K. (2017). Feasibility and outcome of an accelerated recovery protocol in Asian adolescent idiopathic scoliosis patients. Spine.

[76] Charette S., Fiola J.L., Charest M.C., Villeneuve E., Théroux J., Joncas J., Parent S., Le May S. (2015). Guided imagery for adolescent post-spinal fusion pain management: A pilot study. Pain. Manag. Nurs..

[77] LaMontagne L.L., Hepworth J.T., Cohen F., Salisbury M.H. (2003). Cognitive-behavioral intervention effects on adolescents’ anxiety and pain following spinal fusion surgery. Nurs. Res..

[78] LaMontagne L., Hepworth J.T., Salisbury M.H., Cohen F. (2003). Effects of coping instruction in reducing young adolescents’ pain after major spinal surgery. Orthop. Nurs..

[79] LaMontagne L.L., Hepworth J.T., Cohen F., Salisbury M.H. (2004). Adolescent scoliosis: Effects of corrective surgery, cognitive-behavioral interventions, and age on activity outcomes. Appl. Nurs. Res..

[80] Nelson K., Adamek M., Kleiber C. (2017). Relaxation training and postoperative music therapy for adolescents undergoing spinal fusion surgery. Pain. Manag. Nurs..

[81] Rhodes L., Nash C., Moisan A., Scott D.C., Barkoh K., Warner W.C., Sawyer J.R., Kelly D.M. (2015). Does preoperative orientation and education alleviate anxiety in posterior spinal fusion patients? A prospective, randomized study. J. Pediatr. Orthop..

[82] Ying L., Fu W. (2020). The positive role of rosenthal effect-based nursing in quality of life and negative emotions of patien ts with scoliosis. Int. J. Clin. Exp. Med..

[83] Hinrichsen G.A., Revenson T.A., Shinn M. (1985). Does self-help help?. J. Soc. Issues.

