# Supplementary material for: Assessing the Efficacy of Cognitive-Behavioral Therapy on Body Image in Adolescent Scoliosis Patients Using Virtual Reality
Source: J Clin Med. 2024 Oct 26;13(21):6422. doi: 10.3390/jcm13216422 (PMC11546488; doi:10.3390/jcm13216422)
Supplement: Supplementary file 1 [file jcm-13-06422-s001.zip › jcm-3199929-supplementary.pdf]

Material and Methods

Table S1.
Intervention module

Baseline	Meeting the inclusion criteria<br>Recruitment to the study<br>Randomization	
Before the surgery	CBTSS	CSS
	Collecting socio-demographic, clinical, and radiological data	
	Completing VR tasks	
	CBTSS - receives CBT support	CSS - without CBT support
After the surgery	CBTSS	CSS
	Collecting clinical and radiological data	
	CBTSS - receives CBT support	CSS - without CBT support
	Completing VR tasks	

Note CBT SS-CBT scoliosis sample; CSS- control scoliosis sample; HFS- healthy female sample; VR-virtual reality;

Application "*Avatar Scoliosis 3D"*

The application was developed using the Unity engine, version 2019.4.37 (Unity Technologies, San Francisco, CA, USA). VR interactions and camera movements are facilitated through the SteamVR plugin for the Unity engine, version 2.7.3 (Valve Corporation, Bellevue, WA, USA), available on the Unity Asset Store platform and compatible with the SteamVR desktop application (available on the Valve Steam platform, version 1.24), and the Vive Input Utility plugin for Unity Engine, version 1.17.0. These assumptions remained unchanged. However, in the final application, there was a change in the software used to simulate avatar movement (inverse kinematic solver). The Final IK plugin was deemed unsuitable and was replaced by BioIK, version 2.0 (purchased through the Unity Asset Store platform). The BioIK plugin constructed avatars' skeletons with appropriately placed joints in anatomically correct positions. This was done considering the anatomical limits of movement for each joint and ensuring that it was impossible to deform the avatar during the application's use beyond what the human body can achieve. The researchers tested the application thoroughly, as shown in Fig. 2.

Experimental procedures for CBT scoliosis sample and control scoliosis sample

In summary, the "Avatar Scoliosis 3D" application developed an experimental procedure featuring a library of realistic virtual 3D avatars, allowing for realistic body manipulations and presenting these avatars in a naturalistic scenario. Generally, the researcher provided all patients with an explanation of the purpose of the VR tasks using "Avatar Scoliosis 3D," detailing the components of this VR-related task. Participants were informed that the study aimed to examine their perception of actual and desired body shape [52]. Specifically, the procedure included E1 and E2, during which avatars were displayed on an immersive life-size stereoscopic screen, simulating the experience of looking at oneself in a mirror in VR.

Patients could view the selected avatar in motion [52]. They completed two method-of-adjustment tasks (MoA): the first focusing on their current body shape (E1) and the second on their desired body shape (E2) [52]. In both the E1 and E2 parts, patients were shown each avatar referring to the following ranges of Cobb's angle: Avatar no. 1 with a Cobb angle of 10–19; no. 2 with a Cobb angle of 20–29; no. 3 with a Cobb angle of 30–39; no. 4 with a Cobb angle of 40–49; no. 5 with a Cobb angle of 50–59; no. 6 with a Cobb angle of 60–69; and no. 7 with a Cobb angle of 70–79 [52].

The investigator showed instructions at the start of E1 or E2, followed by the avatar's appearance. In E1, participants were instructed: "Please adjust the body shape on screen until it matches your current body!" For E2, the instruction was: "Please adjust the body shape on screen until it matches your ideal, desired body shape." During the session, participants could change the selected avatar to one with a different Cobb angle (lower or higher) if their perception did not align with the initial avatar visualization. The session had no set time limit, but participants were advised to rely on their instincts and spend only a short time on each decision [52].

Due to the specified technical requirements of this application, VR tasks were conducted during the 1st and 2nd study phases at the Department of Pediatric Orthopedics and Traumatology and the Department of Spine Disorders and Pediatric Orthopedics.

Experimental procedures for healthy female sample

We also developed an experimental procedure for healthy controls. We assumed that healthy females in both experimental tasks (E1 and E2) should adjust avatar no. 1. At the beginning of each experiment, the investigator provided the instruction, after which the avatar appeared. In E1, the instruction was: "Please adjust the body shape on screen until it matches your current body!". In E2, the instruction was modified: "Please adjust the body shape on screen until it matches your ideal, desired body shape" [52].

The content of CBT pre- and postsurgical sessions

The presurgical sessions involved discussing emotions and beliefs related to negative body image, as well as exploring the behavioral consequences that negative body image may promote, such as dieting, checking, and avoidance behaviors. Participants also addressed triggers of negative body image, including sociocultural, peer, familial, and health-related factors. Additionally, developmental factors contributing to their body image and immediate sources of body image distress were highlighted during these sessions.

In addition, a presurgical session included writing down and discussing patients' appearance-preoccupying rituals. Examples may consist of frequent inspection in front of a mirror and regular weighing, as well as time-consuming efforts to manage, repair, or alter one's appearance through meticulous grooming routines. In another presurgical session, participants were prompted to write down and discuss their beliefs about what would happen if their distressing body parts were openly revealed. The objective of this exercise was to uncover the personal maladaptive nature of these thoughts, which contribute to dysfunctional body image, feelings, and behaviors. Each assumption's validity was challenged and discussed during these sessions. Patients were also encouraged to consult their compensatory behaviors due to their body image concerns. This discussion aimed to enhance the patient's body relationship by expanding their sense of control over it and fostering enjoyment in caring for their body appropriately.

The length and the content of postsurgical sessions strongly depended on individual patient needs. Concerning the detailed components of CBT postsurgical sessions, they were steered towards the reduction of distressing self-consciousness. The therapist explained that negative thoughts toward one's silhouette are learned and, therefore, can be unlearned, explicitly bearing in mind meaningful visual improvement of body shape following surgical treatment.

Firstly, patients were instructed in relaxation techniques. Secondly, participants learned to replace critical self-talk with more objective sensory descriptions of their corrected parts of the body. The therapist demonstrated corrective body talk using examples provided by the patient. Additionally, thought-stopping and relaxation were introduced to reduce patients' distress. This technique, as described by Thompson [56], involves (a) interrupting negative self-talk in midstream, (b) examining activating events and maladaptive internal body talk to identify cognitive errors contributing to emotional reactions related to body image, and (c) listening to more accurate self-statements that are able correct those errors.

To summarize, the intervention's general components comprised supporting AIS patients in accepting their actual body shape, changing their desired body shape more realistically, and being more satisfied with the cosmetic results of scoliosis surgery.

Statistical methods.

As "Avatar Scoliosis 3D" is a novel methodology, we tested its psychometric properties. Validity was assessed by calculating Spearman's correlation coefficients between the VR tasks (1st indicator: participants' estimated current body shape at the time of E1 and 3rd indicator: participants' actual body shape - based on the radiographic parameters or clinical examination) and the participants' Cobb angle, which served as an objective measure of their actual body shape. Then, as study participants were tested twice via VR tasks, pre- and postoperatively, we calculated test-retest reliability (regarding the 1st and 2nd indicators) using Spearman's correlation coefficients.

Results

Preliminary psychometric properties of "Avatar Scoliosis 3D"

Spearman's correlation coefficients between the 1st and 3rd indicator and the participants' Cobb angle are outlined in Table 3. The coefficients were high and statistically significant in CBTSS and CSS, both pre-a and postoperatively, for the 3rd indicator.

The test-retest reliability of the 1st indicator was average in the CBTSS and moderate in the CSS. Meanwhile, referring to the 2nd indicator, the values were high for the CBTS and CSS, meaning the test results are stable and consistent over time (for details, see Table S2).

Table S2.
Preliminary psychometric properties of "Avatar Scoliosis 3D."

	Preoperatively	Postoperatively
	CBTSS/CSS	CBTSS/CSS
1st indicator & Cobb angle	rs=-0.28/rs=0.21	rs=-0.09/rs=0.08
3rd indicator & Cobb angle	rs=0.93*/rs=0.94*	rs=0.73*/rs=0.89*
	CBTSS	CSS
Preoperative 1st indicator &postoperative 1st indicator	rs=0.52*	rs=0.65*
Preoperative 2nd indicator &postoperative 2nd indicator	rs=0.76*	rs=0.78*

Note: CBT SS-CBT scoliosis sample; CSS-control scoliosis sample; 1st indicator: participants' estimated current body shape at the time of E1; 2nd indicator: participants' desired body shape at the time of E2; 3rd indicator: participants' actual body shape (based on the radiographic parameters or clinical examination; *p<0.05.
